# Effects of body fat mass and therapeutic weight loss on vitamin D status in privately owned adult dogs

**DOI:** 10.1017/jns.2018.7

**Published:** 2018-04-18

**Authors:** Tabitha J. Hookey, Robert C. Backus, Allison M. Wara

**Affiliations:** Department of Veterinary Medicine and Surgery, College of Veterinary Medicine, University of Missouri, Columbia, MO 65211, USA

**Keywords:** Cholecalciferol, 25-Hydroxyvitamin D, Adiposity, Canine weight loss, 24,25(OH)_2_D_3_, 24,25-dihydroxyvitamin D_3_, 25(OH)D, 25-hydroxyvitamin D, 25(OH)D_3_, 25-hydroxyvitamin D_3_, BCS, body condition score, BF, body fat, BF%, body fat percentage, BFM, body fat mass, BLM, body lean mass, BW, body weight, NRC RA, National Research Council recommended allowance

## Abstract

More than one-third of humans and companion dogs in Western societies are overweight or obese. In people, vitamin D deficiency is widespread and associated with obesity, a now recognised inflammatory state. Low vitamin D status occurs in dogs with inflammatory conditions, but its relationship with obesity has not been investigated. In otherwise healthy privately owned adult dogs of ideal body condition (control, *n* 7) and dogs with overweight to obese body condition (treatment, *n* 8), serum 25-hydroxyvitamin D (25(OH)D) concentration and body composition as inferred from ^2^H-labelled water dilution space were evaluated. Subsequently, the dogs were transitioned to a commercial canine therapeutic weight-loss diet; control dogs were fed to maintain body weight and treatment dogs were energy-restricted to achieve a safe weight-loss rate. Thereafter, serum 25(OH)D concentration was re-evaluated 8 weeks after diet transition, and at the study end, which was 6 months or when ideal body condition was achieved. At study end, body composition analysis was repeated. Initial body condition scores and percentage body fat were positively correlated (ρ = 0·891; *P* < 0·001). However, percentage body fat and serum 25(OH)D concentration were not significantly correlated. Final serum 25(OH)D concentrations were greater (*P* < 0·05) than initial concentrations for control and treatment groups, indicating a diet but not weight-loss effect on vitamin D status. These findings suggest that vitamin D status of dogs is not affected by obesity or loss of body fat with therapeutic weight reduction.

In Western societies, obesity is a pervasive disease affecting both human and companion animal populations in similar magnitudes. In 2013–2014, a national survey of 5455 adults conducted in the USA found that nearly 40 % were obese^(^^1^^)^. Several studies have also investigated the prevalence of obesity in populations of companion dogs, reporting like findings with 33·5–38·9 % of dogs classified as overweight^(^^2^^–^^4^^)^. In humans, vitamin D deficiency, which is generally defined as serum 25-hydroxyvitamin D (25 (OH)D) concentration of <20 ng/ml, is also a worldwide health issue^(^^5^^)^. In addition to vitamin D's established roles in Ca homeostasis and skeletal health, a vast body of evidence now describes many extraskeletal physiological effects of vitamin D status in people. Associations have been discovered between hypovitaminosis D and numerous immune-mediated, endocrine, cardiorespiratory, neoplastic and inflammatory diseases^(^^6^^–^^14^^)^. Additionally, though causality has not been elucidated, an association between vitamin D deficiency and human obesity has been documented for decades^(^^15^^–^^18^^)^.

Following trends in human medicine, there too is interest in the extraskeletal effects of vitamin D, and the vitamin's role in health and disease in companion dogs. While vitamin D is an increasingly active area of veterinary medical research, vitamin D sufficiency, the serum 25(OH)D concentration considered adequate for skeletal and general health for the majority of the population, remains undefined in dogs. One group has suggested serum 25(OH)D of 100–120 ng/ml as sufficient^(^^19^^)^. Variable and wide ranges in serum 25(OH)D concentration have been reported in healthy dogs. Additionally, as compared with healthy or unaffected control dogs, low 25(OH)D concentrations have been found in dogs affected with such common canine diseases as protein-losing enteropathy, International Renal Interest Society stages 3 and 4 chronic kidney disease, and congestive heart failure^(^^20^^–^^23^^)^. Several studies have also reported on vitamin D status in dogs with neoplasia as compared with healthy control dogs. Specifically, lower serum 25(OH)D concentrations have been reported in dogs with splenic haemangiosarcoma, cutaneous mast cell tumours, neoplastic spirocercosis and lymphoma^(^^19^^,^^24^^–^^26^^)^.

Despite the prevalence of canine obesity and research demonstrating the occurrence of systemic inflammation in obese dogs, to our knowledge, the relationship between vitamin D status and obesity in dogs has not been investigated^(^^27^^–^^29^^)^. In a recently published study evaluating plasma 25(OH)D concentrations in sixty-nine dogs with cancer and twenty-three healthy control dogs, a significant relationship between body condition score (BCS) and vitamin D status was not found^(^^30^^)^. The majority of dogs in the study were in ideal body condition or overweight, and the number of obese subjects was not described^(^^30^^)^. One goal of the present study was to assess the relationship between adiposity and vitamin D status (as indicated by serum 25-hydroxyvitamin D_3_ (25(OH)D_3_) and 24,25-dihydroxyvitamin D_3_ (24,25(OH)_2_D_3_) concentrations) in otherwise healthy adult dogs in ideal, overweight or obese body condition. A second goal was to evaluate the effect of body fat (BF) loss on vitamin D status in a cohort of overweight adult dogs during therapeutic weight reduction. We hypothesised that vitamin D status and adiposity would be negatively correlated in adult dogs, and that serum 25(OH)D concentrations would increase as overweight dogs lost body fat mass (BFM).

## Materials and methods

### Animals

Lean dogs with an ideal BCS of 4–5/9 and overweight dogs with a BCS of 6–9/9 were recruited for participation in the study. Fifteen adult dogs, aged 1–12 years (median 5 years), privately owned by staff, faculty, and students at the university were volunteered. Three of the overweight dogs were recruited during their participation in a pet weight-loss programme run by veterinary students at the university. The study population was comprised of ten male neutered and five female spayed mixed breed and purebred dogs with body weights (BW) from 4·1 to 43·0 kg (median 18·4 kg) (Table 1). Each dog was deemed clinically healthy at the time of enrolment based on evaluation of findings of physical examination by the same investigator (T. J. H.), complete blood cell count, serum biochemistry profile and urinalysis. Initial BCS and muscle condition scores were assessed by the same investigator (T. J. H.)^(^^31^^,^^32^^)^. Dogs with a BCS of less than 4/9, that were not amenable to handling, that were clinically ill, or that weighed less than 4·0 kg were excluded from the study. The owners of participating dogs completed a questionnaire detailing each dog's diet history, activity level and home environment. The owners consented to dietary modification with the study diet and agreed to follow the provided feeding instructions for the duration of the study. The study protocol was reviewed and approved by the University of Missouri Institutional Animal Care and Use Committee. The owners of participating dogs were required to sign an informed consent form prior to inclusion in the study.

**Table 1.** Demographics of dogs in the control and treatment groups (Medians and ranges)

	Group			
	Control (*n* 7)		Treatment (*n* 8)	
	Median	Range	Median	Range
Body weight (kg)	18·4	4·1–30·2	20·7	7·0–43·0
Age (years)	5	1–12	5·5	3–10
Body condition score (nine-point scale)	5	4–5	8	6–8
Sex				
Female spayed (*n*)	3		2	
Male neutered (*n*)	4		6	
Breed	Brittany spaniel (*n* 1), German shepherd (*n* 1), miniature pinscher (*n* 1), Pomeranian (*n* 1), mixed breed (*n* 3)		Australian shepherd (*n* 1), beagle (*n* 1), Cairn terrier (*n* 1), golden retriever (*n* 1), rough coated collie (*n* 1), mixed breed (*n* 3)	

### Experimental design

Prior to transition to the study diet and following overnight food withholding, blood (5 ml) was collected from all dogs by venepuncture from a peripherally accessible vein, and serum of the blood extracted. Serum was stored (−20°C) for analysis of vitamin D metabolites by HPLC at a later date. Aliquots of the same serum samples were later used to determine initial body composition using a ^2^H-labelled water dilution method. Subsequently, all dogs were gradually transitioned to the study diet over a period of approximately 7 d. Seven lean dogs with ideal BCS of 4–5/9 were assigned to the control group for which weight maintenance was the goal. Owners of dogs in the control group were instructed to maintain their dogs’ historical daily metabolisable energy (ME) intake on the study diet, and were provided with written feeding orders for achieving this. Eight overweight or obese (BCS of 6–9/9) dogs were assigned to the treatment group for which weight loss was the goal. Owners of dogs in the treatment group were instructed to reduce their dogs’ daily energy intake to achieve weight loss at the desired rate of 1–2 % of BW weekly^(^^33^^)^ and were provided with written feeding orders. To aid in owner compliance, all dogs were provided a maximum allowance of 10 % of total daily energy intake from food items other than the study diet. Following transition to the study diet, each dog was weighed in 2- to 4-week intervals to determine the need for adjustment in energy intake based on the goal of weight maintenance or loss. Body and muscle condition scores were reassessed by the same investigator (T. J. H.) at weigh-ins, or as deemed appropriate. Approximately 8 weeks after transition to the study diet and after overnight food withholding, venous blood (5 ml) was collected for repeated analysis of serum vitamin D metabolite concentrations. For the aforementioned three dogs in the treatment group only, this was the initial blood collection. Dogs in the control group were intended to remain on the study diet for approximately 6 months. Overweight dogs were committed to remain on the study diet for approximately 6 months, or until a BCS of 4–5/9 was attained. At the end of the study and after overnight food withholding, venous blood (5 ml) was collected from dogs of both control and treatment groups for a final determination of serum vitamin D metabolite concentrations and repeated body composition analysis using the same methods.

### Diet

The study diet was a commercially available therapeutic, canine weight-loss, dry-type diet that had undergone animal feeding tests using Association of American Feed Control Officials (AAFCO) procedures to demonstrate nutritional adequacy for the maintenance of adult dogs (Purina® Pro Plan® Veterinary Diets OM Overweight Management™ Canine Formula; Nestlé Purina). The diet was characteristic for use in weight loss in that compared with diets typically available to consumers for maintenance of dogs, it was lower in energy density and fat, and higher in protein and fibre. Manufacturer-reported nutrient profiles of the study diet are provided in Table 2. The study diet was believed to be sourced from multiple batches based on differing lot numbers displayed on the product packaging. To determine batch variability in vitamin D content, four samples collected from different batches of the study diet were analysed for cholecalciferol (vitamin D_3_) and ergocalciferol (vitamin D_2_) contents at an external laboratory (Eurofins Nutrition Analysis Center, Des Moines, IA, USA).

**Table 2.** Nutrient profile of the study diet and formula of the study diet briefly consumed by one dog in the treatment group (diet B) as provided by the manufacturer

	Study diet: ME = 10980 kJ/kg (2624 kcal/kg)			Diet B: ME = 11340 kJ/kg (2710 kcal/kg)		
	Per 418 kJ (100 kcal) ME (g)	As fed (%)	DM (%)	Per 418 kJ (100 kcal) ME (g)	As fed (%)	DM (%)
Protein	10·29	27·00	29·64	9·96	27·00	29·70
Fat	2·48	6·52	7·16	2·23	6·03	6·63
Carbohydrate	15·70	41·19	45·22	15·42	41·80	45·98
Crude fibre	3·85	10·11	11·10	3·49	9·46	10·41
Total dietary fibre	8·38	22·00	24·15	8·12	22·00	24·20
Soluble fibre	0·53	1·40	1·54	0·92	2·50	2·75
Insoluble fibre	7·85	20·60	22·62	7·20	19·50	21·45
Ca	0·43	1·12	1·23	0·37	1·00	1·10
P	0·32	0·85	0·93	0·31	0·84	0·92
K	0·28	0·74	0·82	0·44	1·19	1·31
Na	0·07	0·20	0·22	0·10	0·27	0·30
Cl	0·12	0·31	0·34	0·16	0·44	0·48
Mg	0·06	0·15	0·16	0·07	0·20	0·22
Cu	0·66 mg	17·4 mg/kg	19·1 mg/kg	0·69 mg	18·6 mg/kg	20·4 mg/kg
Zn	10·10 mg	265 mg/kg	291 mg/kg	9·70 mg	263 mg/kg	289 mg/kg
Vitamin A	1305 IU	34 250 IU/kg	37 604 IU/kg	1040 IU	28 184 IU/kg	31 000 IU/kg
Vitamin E	20·01 IU	525 IU/kg	576 IU/kg	16·27 IU	441 IU/kg	485 IU/kg
Cholecalciferol	55·8 IU		1607 IU/kg	41·6 IU		1317 IU/kg
EPA + DHA	0·00	0·00	0·00	0·004	0·01	0·01

ME, metabolisable energy.

### Serum 25-hydroxyvitamin D metabolites

Serum 25(OH)D_3_ and 24,25(OH)_2_D_3_ concentrations were determined in our laboratory using modifications of previously described extraction and HPLC methods^(^^34^^)^. Thawed serum samples (0·5 ml) were spiked with 25 ng of internal standards, 25(OH)D_2_ and 3-epi-24,25(OH)_2_D_3_ (Isosciences), vortex-mixed and incubated for 15 min at room temperature. Acetonitrile (0·5 ml) was added to each sample followed by centrifugation (20°C; 2000 ***g***; 10 min). The supernatant was removed and added to tubes containing doubly distilled water (0·5 ml). The tube contents were vortex-mixed, and loaded on cartridges containing 500 mg of C18 silica gel (Bond Elut; Agilent Technologies) after sequential conditioning with hexane, isopropanol, methanol, and water (2 ml each). The cartridges were washed with water (5 ml), methanol–water (70:30, v/v; 5 ml), and air-dried (10 min). Following air-drying, the 25(OH)D on cartridges was eluted with hexanes–methylene chloride (90:10, v/v; 5 ml) and the 24,25(OH)D eluted with hexanes–isopropanol (95:5, v/v; 5 ml). The eluents were combined and reduced to dryness by centrifugal evaporation at 35°C (Savant SPD 111 V; Thermo Electron). Residues were dissolved in HPLC mobile phase (hexanes–isopropanol, 88:12, v/v) and injected (0·2 ml) on a column (Zorbax Sil, 4·6 × 250 mm, 5 µm; Agilent Technologies) equilibrated with the mobile phase flowing at 2 ml/min. Eluting normal-phase fractions containing 25(OH)D and 24,25(OH)_2_D extracted from serum were collected and dried by centrifugal evaporation. After reconstitution in 0·15 ml methanol–water (67:33, v/v), the metabolites were quantified by the reverse-phase HPLC method described by Lensmeyer *et al.*^(^^35^^)^. For this, 0·1 ml of reconstitute was injected into the mobile phase of methanol–water (67:33 v/v for 25(OH)D; 62:38 v/v for 24,25(OH)_2_D_3_) flowing at 1·2 ml/min through a cyanopropyl column (Zobax SB-CN, 4·6 × 250 mm, 5 µm; Agilent Technologies) heated to 50°C. AUC absorbances at 265 nm of eluting analyte and internal standard peaks were proportional to the amount of vitamin D metabolite injected. The lower limit of metabolite quantification was determined to be the amount equivalent to 5 ng/ml of the metabolite in serum.

### Body composition

To quantify BFM and body lean mass (BLM), we used a modification of a previously validated ^2^H-labelled water dilution method^(^^36^^)^. After overnight food withholding but with water provided to the dogs for *ad libitum* consumption, BW were determined and ^2^H-labelled water (99·8 %; Acros Organics) as a sterile saline solution (0·9 % NaCl) was administered to each dog subcutaneously at 0·4 g/kg BW. At 3 h later, peripheral venous blood was collected for extraction of serum, which was in turn stored at −20°C until later analyses. Upon thawing, water was distilled from aliquots of sampled serum^(^^37^^)^ and its ^2^H-labelled water enrichment determined using infrared spectroscopy^(^^38^^)^. Mass of body water was determined by dividing the amount (g) of subcutaneously administered ^2^H-labelled water by the ^2^H-labelled water enrichment in serum water (g/kg)^(^^39^^)^. Lean mass of dogs was estimated as the mass of body water divided by the fractional moisture content of BLM, which across species is conventionally assumed to be 0·732^(^^40^^)^. Fat mass of dogs was taken to be BW minus the estimated lean mass. The timing of equilibration of subcutaneously injected ^2^H-labelled water was determined in a prior trial. In this trial, injected ^2^H-labelled water was salinated and given at the same dosage as that described in the present study to a cohort of university-owned 4-year-old mixed-breed dogs, five of which were male (ranging from 7·1 to 11·2 kg), and five of which were female (ranging from 5·4 to 7·7 kg) . The ^2^H-labelled water enrichment in water extracted from sampled saliva was determined immediately before ^2^H-labelled water administration and hourly for 6 h afterward. Enrichment of ^2^H-labelled water in salivary water among the dogs was found to plateau beginning 3 h after the ^2^H-labelled water administration (R. C. B., unpublished results, 2015).

### Statistical analysis

Commercially available statistical software was used for analyses of effects of experimental group and sampling time on variable observations (SAS^®^ 9.4; SAS Institute Inc.). Evaluated variables were serum concentrations of 25(OH)D_3_ and 24,25(OH)_2_D_3_, BCS, BW, and amounts BLM, BFM and percentage BW as fat (BF%). Deviations from normality in statistically evaluated observations were determined using Shapiro–Wilk testing. Among variable observations significantly deviating from normality were BCS, final BFM and serum 24,25(OH)_2_D_3_ concentration. Wilcoxon two-sample testing was used to determine the significance of experiment group differences involving non-normal observations. Sign tests were used for determination of significance of change in the observations with time. For normally distributed variable observations, significances of differences between the experiment groups and changes with time were determined with two-sample *t* testing and paired *t* testing, respectively, or repeated-measures ANOVA. Linear regression analysis was used to test the significance of correlation between variable observations and serum 25(OH)D_3_ concentration and change in the concentration during the study. Significance of correlation between BF% and BCS was determined using Pearson analysis. Significance of statistical testing outcomes was concluded when analyses indicated a probability of a type I error as less than 5 %. The number of dogs initially recruited for inclusion in the control and treatment groups was based on results of a prior power analysis in which variance in serum concentrations of 25(OH)D_3_ of dogs was determined^(^^41^^)^. A treatment effect resulting in a mean difference of 25 % in serum 25(OH)D_3_ concentration between groups was expected detectable as significant with α = 0·05, β = 0·80, and six to seven dogs in experimental groups.

## Results

**Fig. 1.**
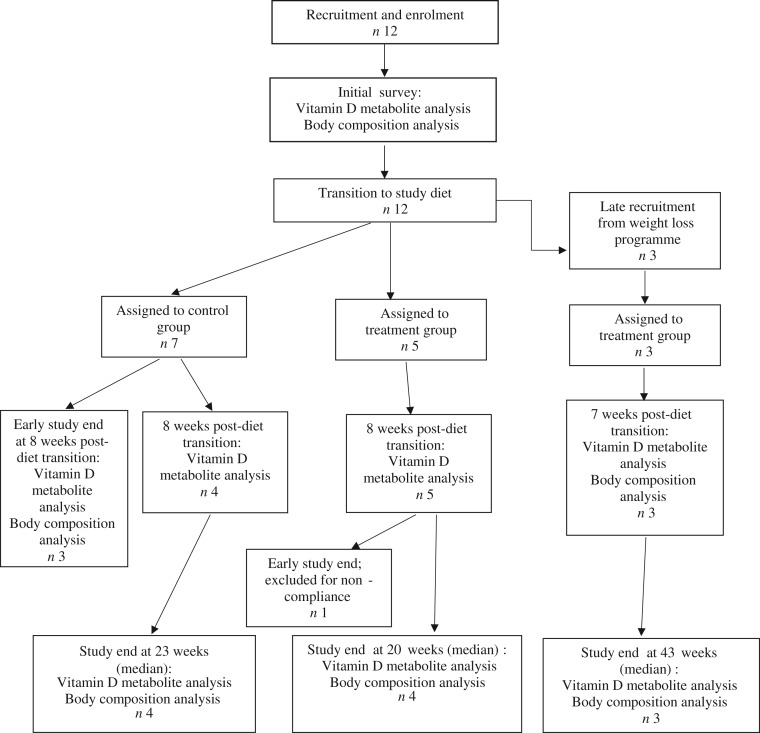
Experimental design and flow of participants through the study period.

Fifteen dogs were enrolled in the study, with seven assigned to the control group, and eight assigned to the treatment group (Fig. 1). Due to owner scheduling conflicts, three dogs in the control group completed the study early after collection of the second serum sample; thus, their second serum samples, collected approximately 8 weeks post-diet transition, were considered their final sampling. For the remaining four dogs in the control group, the median study end was 23 (range 16–28) weeks after diet transition. One dog from the treatment group received food other than that expected for a sustained period after collection of the second serum sample; therefore, only initial body composition observations and initial and 8-week vitamin D metabolite observations for this dog were included in analyses. For the remaining seven dogs in the treatment group, the median study end was 27 (range 15–46) weeks after diet transition.

For a short but undefined period following transition to the study diet, one dog from the treatment group was unintentionally fed a different formula of the study diet (referred to in Table 2 as Diet B; Purina® Pro Plan® Veterinary Diets OM Select Blend Overweight Management™ Canine Formula; Nestlé Purina). On recognition of this error, the dog was changed to the correct study diet for the remainder of the study.

### Analysis of vitamin D content in study diet

The study diet samples analysed contained less than 40 IU of ergocalciferol per kg DM. The cholecalciferol content of three study diet samples ranged from 1580 to 1910 IU per kg DM. These values closely agreed with the manufacturer-reported cholecalciferol content of 1607 IU per kg DM. A sample of diet B contained 944 IU of cholecalciferol per kg DM; the manufacturer reported cholecalciferol content of this formula to be 1317 IU per kg DM.

### Body condition score, body weight and body composition analyses

Median initial BCS of dogs assigned to the treatment group was consistent with the dogs being overweight in condition; it was greater (*P* < 0·001) than that of the control group, which was in agreement with the control dogs being of ideal body condition. Between initial and final observations, BCS of the dogs in the control group did not significantly change, and BCS of the dogs in the treatment group decreased (*P* < 0·01). Final BCS of dogs of the treatment group remained significantly greater (*P* < 0·05) than dogs of the control group.

Initial BW of treatment and control group dogs were not significantly different (Table 3). BW of dogs of the control group did not significantly change between initial and final observations. Final BW of dogs of the treatment group were significantly less than the initial BW (*P* < 0·01).

**Table 3.** Body condition score, body weight, and body composition observations for control and treatment group dogs (Medians and ranges)

	Initial				Final			
	Control (*n* 7)		Treatment (*n* 7*)		Control (*n* 7)		Treatment (*n* 7)	
	Median	Range	Median	Range	Median	Range	Median	Range
Body condition score (nine-point scale)	5	4–5	8†	6–8	4	4–5	5†‡	4–7
Body weight (kg)	18·4	4·1–30·2	18·3	7·0–43·0	17·4	4·0–30·9	15·5^‡^	5·8–36·0
Lean body mass (kg)	13·0	3·3–22·2	12·5	4·0–26·8	13·1	3·3–23·1	10·9^‡^	3·6–26·6
Fat body mass (kg)	5·3	0·8–8·0	6·9	3·0–20·5	4·3	0·7–7·8	4·6^‡^	1·8–14·5
Percentage body fat (%)	26·1	18·6–29·2	39·6^†^	31·4–48·1	25·1	17·2–33·8	29·5†‡	23·6–40·4

* Observations for one treatment group dog for which only initial body composition analyses were available have been excluded.

† Observations significantly different from control observations at the same time (*P* < 0·05).

‡ Observations significantly different from corresponding initial group observations (*P* < 0·05).

Body composition data were collected at the time of study enrolment and again at the end of the study of each dog. Between groups of dogs, there was no significant difference in BLM or BFM at the initial observation (Table 3). The BLM decreased in the treatment group over time (*P* < 0·01). The BFM of the treatment group decreased (*P* = 0·01), whereas the BFM of the control group did not significantly change. The treatment group had a median initial BF% of 39·6 %, which was higher than that of the control group (median of 26·1 %) (*P* < 0·001). By the end of the study, BF% was decreased in dogs of the treatment group (*P* < 0·001), among which final median BF% was 29·5 %. For all dogs, initial BCS was correlated with initial BF% estimates (ρ = 0·891; *P* < 0·001).

### Serum 25-hydroxyvitamin D_3_ and 24,25-dihydroxyvitamin D_3_

**Fig. 2.**
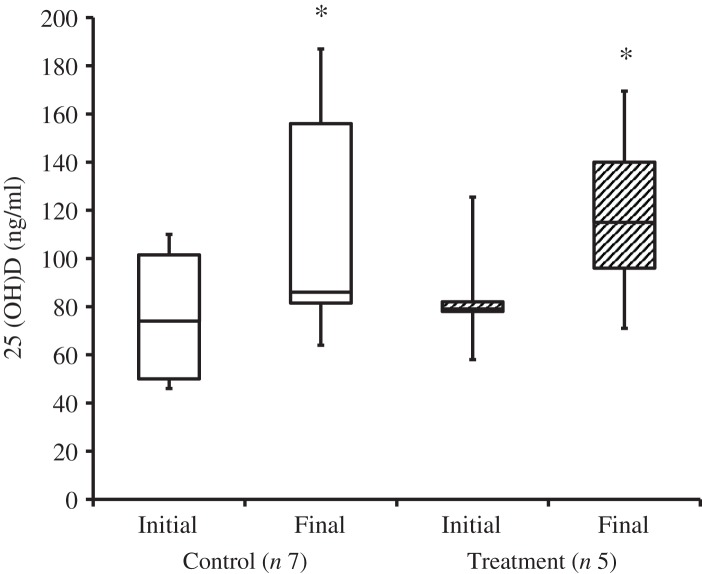
Serum 25-hydroxyvitamin D (25(OH)D) concentrations of dogs in the control and treatment groups, including initial observations (prior to transition to study diet) and final observations at study end. Each plot represents observations' median (centre line), first quartile (lower box line), third quartile (upper box line), minimum (lower whisker) and maximum (upper whisker). * Observations significantly different from corresponding initial group observations (*P*<0.05).

At the time of study enrolment prior to dietary transition, serum vitamin D metabolite concentrations were determined in twelve dogs. At the initial observation, all dogs were consuming commercially available canine diets. Most of the dogs were maintained on a commercial dog food other than the study diet, with the exception of the three additional dogs later assigned to the treatment group which were already consuming the study diet. For these three dogs only, initial blood sampling for analyses of serum vitamin D metabolite concentrations occurred after approximately 7 weeks of exclusive consumption of the study diet, and these initial observations were excluded from analysis of effects of dietary transition. For the other twelve dogs, serum vitamin D metabolite concentrations were re-evaluated approximately 8 weeks after dietary transition; this 8-week point was considered the study end for three control dogs that were withdrawn early, and one treatment dog that was excluded for non-compliance thereafter. Finally, serum vitamin D metabolites were again measured in four dogs in the control group and all remaining seven dogs in the treatment group at study end. The median initial serum concentrations of 24,25(OH)_2_D_3_ were not significantly different between the control (57 ng/ml) and treatment (75 ng/ml) groups. For both groups, serum 24,25(OH)_2_D_3_ concentrations did not change between initial and final observations. The median initial serum 25(OH)D_3_ concentration was 74 (range 46–110) ng/ml for the control group, and 79 (range 53–129) ng/ml for the treatment group; these were not statistically different. As compared with the corresponding initial group observations, final median serum 25(OH)D_3_ concentrations of dogs in both the control and treatment groups, which were 86 (range 64–187) and 115 (range 89–163) ng/ml, respectively, were significantly (*P* = 0·02) increased (Fig. 2).

When the initial observations from dogs of control and treatment groups were pooled, BF% and serum 25(OH)D_3_ concentration were not significantly correlated (*r* 0·11; *P* = 0·69). Furthermore, when diet was controlled, that is when only the study diet was fed in amounts required for safe weight loss, fractional loss of BFM and decrease in BF% in dogs of the treatment group (*n* 7) did not significantly correlate with a change in serum 25(OH)D_3_ concentration (*r* 0·15, *P* = 0·74 and *r* 0·56, *P* = 0·19, respectively).

### Effect of daily cholecalciferol intake on serum 25-hydroxyvitamin D_3_

For four dogs in the control group and seven dogs in the treatment group that remained in the study for greater than 8 weeks post-diet transition, daily cholecalciferol intake at the 8-week post-diet transition point was compared with daily cholecalciferol intake at the study end. While changes were observed, there was not a significant correlation between the fractional change in daily cholecalciferol intake and the change in serum 25(OH)D_3_ concentrations (*r* 0·032; *P* = 0·90). Three of the four dogs in the control group had no change in their daily cholecalciferol intake. One dog of the control group required a 33 % increase in energy intake to maintain ideal BW; thus, this dog's cholecalciferol intake increased by 33 %. Of the seven dogs remaining in the treatment group, one of these dogs had no change in its cholecalciferol intake. Another one of these dogs required an energy increase of 52 % to halt weight loss and stabilise BW; thus, this dog's cholecalciferol intake increased by 52 %. The remaining five dogs of the treatment group were consuming a median of 32 (range 1–49) % less cholecalciferol at the end of the study as compared with the time of 8 weeks post-diet transition.

## Discussion

The results of our study do not support degree of adiposity as influencing the vitamin D status of apparently healthy adult dogs. In dogs and other species, the concentration of the vitamin D metabolite 25(OH)D_3_ in peripheral venous serum is conventionally used as an indicator of vitamin D status^(^^42^^)^. Across all the dogs presently studied, initial serum 25(OH)D_3_ concentrations were not significantly correlated with BF%. Also, loss of adiposity in study dogs as a result of energy restriction for therapeutic weight loss did not significantly affect serum 25(OH)D_3_ concentration.

The causality of the inverse relationship between vitamin D status and adiposity that occurs in humans remains unknown^(^^15^^–^^18^^)^. Vitamin D is fat-soluble and stored in adipose tissue. One predominant hypothesis in the human literature is that vitamin D is sequestered in the greater BFM of obese individuals as compared with lean cohorts, and that this leads to reduced bioavailability and circulating serum concentrations of 25(OH)D^(^^43^^)^. Previous studies have measured vitamin D concentrations in fat deposits of people and rats, which are supportive of adipose tissue as a storage site for the vitamin^(^^44^^,^^45^^)^; however, the author is not aware of such studies in dogs. Though seemingly unlikely, it is possible that the distribution of vitamin D stores in the canine is unique for this species. More recently, in contrast to the theory of vitamin D sequestration, another group proposed that volumetric dilution is responsible for lower serum 25(OH)D levels in obese humans, and that cholecalciferol is simply distributed in the total BFM, which is greater in such individuals^(^^46^^)^. It has also been suggested that obesity may be a consequence of hypovitaminosis D and that the vitamin plays complex regulatory roles in adipose, though some studies have demonstrated conflicting results and are inconclusive^(^^47^^)^.

The present study population was comprised of a variety of dog breeds. A previous study of golden retriever and German shepherd dogs found that the latter had a 26 % higher median serum 25(OH)D concentration^(^^48^^)^. In this prior study, however, diet was not controlled and vitamin D intake of individual dogs was unknown. Additionally, Sharp *et al.*^(^^48^^)^ found that a sexually intact status affected the dogs’ serum 25(OH)D concentration, a variable which was not controlled in their study. Differences in serum 25(OH)D concentration have been described in growing great Danes and miniature poodles; however, growth stage of the dogs studied also differed^(^^49^^)^. Therefore, while it is conceivable that breed may affect vitamin D metabolism in dogs, this remains unknown. Age was not controlled in our study dogs, and ranged from young adult (1 year) to senior (12 years). Advancing age is a risk factor for vitamin D deficiency in people for reasons other than reduced sun exposure^(^^50^^)^. A significant correlation between age and serum 25(OH)D has not been definitively demonstrated in dogs. Weidner *et al.*^(^^30^^)^ did not observe a correlation between serum 25(OH)D concentration and age in a population of predominantly older dogs (≥6 years), but described a trend toward significance between the two variables. In another study of 320 dogs aged 0·4–14·5 years, a significant association between age and serum 25(OH)D concentration was not identified^(^^48^^)^. Studying thirty-nine dogs aged 6 months to 11·3 years, Titmarsh *et al*.^(^^51^^)^ also did not find age to be a predictor of serum 25(OH)D. Future studies are needed to determine if age and breed affect vitamin D metabolism of dogs.

A state of chronic inflammation has been observed in obese humans. In veterinary medicine, research has demonstrated inflammatory biomarkers in obese dogs, suggesting that low-grade inflammation may be a component of the condition in this species, too^(^^27^^–^^29^^)^. Furthermore, these reports provide evidence for a reduction in systemic inflammation following weight loss in obese dogs^(^^27^^,^^28^^)^. Vitamin D is considered an immunomodulatory negative acute-phase reactant^(^^42^^,^^52^^)^. Human studies have demonstrated the association of hypovitaminosis D and increased inflammatory biomarkers in obese individuals^(^^53^^)^. The relationship between vitamin D status and systemic inflammation in dogs is less clear. In a group of twelve sled-racing dogs, increased serum C-reactive protein concentrations were associated with increased serum 25(OH)D concentrations during strenuous exercise^(^^54^^)^. One study examining dogs with chronic enteropathy found a negative correlation between serum 25(OH)D concentrations and both leucocyte counts and IL-2 and IL-6 concentrations^(^^51^^)^. Another group reported decreasing C-reactive protein concentrations in dogs as serum 25(OH)D concentrations reached the level they considered sufficient^(^^19^^)^. Clearly, additional work is needed to understand the relationship between systemic inflammation and vitamin D status in dogs. While we did not measure such inflammatory biomarkers in the present study, previous work supports the assumption that these would be present in our population of obese dogs. With the opinion that fat loss would reduce systemic inflammation, we hypothesised in the present study that serum 25(OH)D concentrations would increase as overweight dogs lost BF mass. All dogs in our treatment group that completed the study lost weight and 71 % of these dogs reached an ideal BCS by the study end. However, when diet was removed as a variable, the change in serum 25(OHD) concentration did not reach significance for either group. The dogs in our treatment group underwent 27 weeks (median) of therapeutic weight loss. Over this timespan, the median BF% in the treatment group decreased from 39·6 % to 29·5 %, a reduction of 10·1 % BF. In a study of forty-seven adult humans undergoing weight loss through 15 % dietary energy restriction for 4 weeks, Ibero-Baraibar *et al.*^(^^55^^)^ reported a BF% reduction of 3·38 % (as measured by dual-energy X-ray absorptiometry), associated with significant increases in the 25(OH)D concentrations of the subjects. Based on these results, we might consider the duration of our study and BF% lost to be adequate. While we did not measure BF mass of the dogs after 4 weeks of weight loss, the results of Ibero-Baraibar *et al.*^(^^55^^)^ suggest more rapid BF loss than we probably achieved in the present study. Results of other human studies have suggested that significant increases in vitamin D status do occur with slower fat loss^(^^56^^,^^57^^)^. To our knowledge, whether the rate of fat loss affects change in vitamin D status is unknown.

The distribution of BF stores in overweight dogs may vary among individuals, and may differ from that of obese humans. It is possible that such differences could affect vitamin D status in an obese condition and during fat loss. Subcutaneous and visceral adipose tissue secrete different profiles of inflammatory markers in dogs^(^^58^^)^. A previous study evaluated the distribution of adipose in three adult dogs of different breeds using computed tomography (CT) at 1 year old (at the time of castration) and again 1 year later^(^^59^^)^. BF% increased in the dogs, with the rate of increase of subcutaneous BF significantly higher than thoracic or abdominal adipose, though the study population was limited in age and size^(^^59^^)^. On CT scans of seventeen overweight and obese dogs of different breeds, Thengchaisri *et al.*^(^^60^^)^ found greater amounts of subcutaneous fat as compared with intra-abdominal fat. Conversely, others reported that visceral fat accumulated more quickly than subcutaneous fat in a small group of beagles^(^^61^^)^. Recently, Gangloff *et al.*^(^^62^^)^ found that compared with loss of subcutaneous fat, loss of visceral adiposity had the greatest positive effect on 25(OH)D concentration in 103 adult men. Based on this information, it is conceivable that the distribution of adiposity in our subjects may have influenced the findings of the present study.

Consumption of the study diet appeared to lead to a significant increase in serum 25(OH)D concentration across both groups in our study. Dogs rely almost entirely on dietary intake of vitamin D and lack substantial cutaneous synthesis of the hormone in response to sunlight; this is believed to be a result of high activity of the enzyme 7-dehydrocholesterol Δ^7^-reductase^(^^63^^,^^64^^)^. Over the study period, the majority (64 %) of dogs required a change in energy intake to achieve weight loss or maintenance; some such changes were substantial. However, the fractional changes in energy (and thus, vitamin D_3_) intake were not correlated with the change in serum 25(OH)D_3_ concentration, meaning that increasing serum 25(OH)D concentrations cannot simply be attributed to increased cholecalciferol consumption over time. Our results agree with previous findings that diet has an effect on serum 25(OH)D concentration in dogs^(^^48^^)^. The vitamin D content of the test diet is not extraordinary; it exceeds the National Research Council's recommended allowance (NRC RA) for dietary vitamin D for maintenance of adult dogs, but is well below the safe upper limit^(^^65^^)^. Furthermore, when cholecalciferol intake was compared with the NRC RA for each dog (on a metabolic BW basis), intake ranged from 1·5–4·4 times the NRC RA (8 weeks after dietary transition) to 1·3–4·4 times the NRC RA (at study end). A recent study found that cholecalciferol supplementation of dogs at 5·1 times the NRC RA (which is just below the safe upper limit) affected a modest 12 % increase in vitamin D status in a cohort of dogs^(^^41^^)^. While it is possible that fat loss does not make an impact on vitamin D status in dogs, another possibility is that the effect of the diet on increasing vitamin D status in the study population may have masked a smaller weight-loss effect. Interestingly, though it has been proposed that serum 24,25(OH)_2_D_3_ concentrations, which are positively correlated with 25(OH)D concentrations, may be a useful marker of vitamin D status in people^(^^66^^)^, our results did not indicate a significant corresponding increase in concentrations of 24,25(OH)_2_D_3_ in dogs. This finding was surprising, as 24,25(OH)_2_D_3_ is expected to occur in direct proportion to 25(OH)D_3_ concentration. Investigation of 24,25(OH)_2_D_3_ functionality in dogs is warranted.

BCS was correlated with BF%, consistent with previous findings^(^^31^^,^^67^^)^. In the present study, a single individual (T. J. H.) assessed BCS, and the assigned scores slightly underestimated the BF% of dogs in both groups. Agreement of BCS and objectively measured BF may have been improved by having multiple investigators assess BCS of the subjects. While it is also plausible that body composition analyses using ^2^H-labelled water dilution were inaccurate due to the assumptions that are inherent to this method, previous work has demonstrated agreement between objective measures of BF (such as ^2^H-labelled water dilution and dual-energy X-ray absorptiometry) and the semi-quantitative BCS system for dogs^(^^67^^)^. We considered the reduced procedural cost and lack of required general anaesthesia for the ^2^H-labelled water dilution method to be advantageous. However, in future studies, use of the conventional standard method of body composition analysis, dual-energy X-ray absorptiometry, may be considered.

The present study has several limitations. Dogs were considered clinically healthy based on physical examination findings and clinicopathological data. In the absence of an exhaustive medical workup, it is possible that some subjects had subclinical diseases that may have affected vitamin D metabolism. In addition, given the use of client-owned pet dogs in this study, the owners were relied upon to accurately quantify and dispense food according to the provided instructions. It is possible that the energy intake of the dogs (and thus vitamin D intake) was higher or lower than our calculations. Additionally, dogs may have consumed food items containing vitamin D that owners were not aware of, or those that went unreported. Three dogs consumed consistent quantities of dietary fish oil supplements for the duration of the study, which may have contained cholecalciferol, a known nutrient contained in fatty fish^(^^68^^)^. One such dog, assigned to the treatment group, was given 2000 mg of anchovy and sardine oil per d. Prior to dietary transition, the dog's serum 25(OH)D concentration was 78 ng/ml. As the median initial serum 25(OH)D concentration of both the pooled groups and the treatment group was found to be 79 ng/ml, the supplement is not suspected to have contained a substantial amount of cholecalciferol. The other two dogs, which shared a household and were both assigned to the control group, consumed a product with fish oil of unknown origin. One of these dogs consumed 4000 mg of fish oil daily; the dog's initial serum 25(OH)D concentration was 52 ng/ml. The other dog was consuming 2000 mg of fish oil per d; the dog's initial serum 25(OH)D concentration was 98 ng/ml. None of the fish oil supplements was tested for cholecalciferol or 25(OH)D content, and this information may have been useful. Four dogs consumed joint health supplements for the duration of the trial. On review of the ingredients of the supplements, there were no obvious sources of vitamin D disclosed by the manufacturers; however, the possibility of a proprietary ingredient cannot be excluded. None of these dogs supplemented with joint health supplements had unexpectedly high serum 25(OH)D levels as compared with cohorts. A previous study did not find significant differences between median serum 25(OH)D levels in dogs receiving fish oil supplements or fortified dog biscuits as compared with those that were not. However, in the same study, dogs consuming salmon oil supplements had significantly higher serum 25(OH)D levels than dogs not consuming dietary supplements^(^^48^^)^. Finally, while we determined the study to be sufficiently powered to detect significance of a modest treatment effect, our sample size was small, and this is a limitation to extrapolating our findings of a null effect of adiposity on vitamin D status of dogs in general.

In conclusion, the correlation we found between canine BCS and BF% is consistent with previously described work. Our study supports that diet has an effect on serum 25(OH)D concentration in dogs, as has been demonstrated by others. Our data did not support our hypotheses that an inverse relationship exists between body adiposity and vitamin D status in dogs, and that loss of BF would lead to increasing serum 25(OH)D concentrations in overweight dogs undergoing weight loss. Additional studies investigating larger sample sizes in which diet, age and breed are controlled, and inflammatory markers are measured, are warranted to further our understanding of whether relationships between obesity, inflammation and vitamin D status occur in companion dogs.
